# Physiological profile of undifferentiated bovine blastocyst-derived trophoblasts

**DOI:** 10.1242/bio.037937

**Published:** 2019-04-05

**Authors:** Viju Vijayan Pillai, Luiz G. Siqueira, Moubani Das, Tiffany G. Kei, Lan N. Tu, Anthony W. Herren, Brett S. Phinney, Soon Hon Cheong, Peter J. Hansen, Vimal Selvaraj

**Affiliations:** 1 Department of Animal Science, College of Agriculture and Life Sciences, Cornell University, Ithaca, NY 14853, USA; 2 Department of Animal Sciences, University of Florida, Gainesville, FL 32611, USA; 3 Brazilian Agricultural Research Corporation - Embrapa Gado de Leite, Juiz de Fora, Minas Gerais 36038-330, Brazil; 4 Genome Center, Proteomics Core Facility, University of California, Davis, CA 95616, USA; 5 Department of Clinical Sciences, College of Veterinary Medicine, Cornell University, Ithaca, NY 14853, USA

**Keywords:** Trophoblast, Blastocyst, Stem cells, Implantation, Placenta, Pregnancy

## Abstract

Trophectoderm of blastocysts mediate early events in fetal-maternal communication, enabling implantation and establishment of a functional placenta. Inadequate or impaired developmental events linked to trophoblasts directly impact early embryo survival and successful implantation during a crucial period that corresponds with high incidence of pregnancy losses in dairy cows. As yet, the molecular basis of bovine trophectoderm development and signaling towards initiation of implantation remains poorly understood. In this study, we developed methods for culturing undifferentiated bovine blastocyst-derived trophoblasts and used both transcriptomics and proteomics in early colonies to categorize and elucidate their functional characteristics. A total of 9270 transcripts and 1418 proteins were identified and analyzed based on absolute abundance. We profiled an extensive list of growth factors, cytokines and other relevant factors that can effectively influence paracrine communication in the uterine microenvironment. Functional categorization and analysis revealed novel information on structural organization, extracellular matrix composition, cell junction and adhesion components, transcription networks, and metabolic preferences. Our data showcase the fundamental physiology of bovine trophectoderm and indicate hallmarks of the self-renewing undifferentiated state akin to trophoblast stem cells described in other species. Functional features uncovered are essential for understanding early events in bovine pregnancy towards initiation of implantation.

## Abstract (precis)

**Summary:** Fundamental physiology of the bovine trophectoderm and hallmarks of the self-renewing undifferentiated ‘trophoblast stem cells’ are interpreted through systematic analysis of the cellular proteome and transcriptome.

## INTRODUCTION

During initial steps of embryogenesis, trophoblasts emerge as first to commit to a tissue lineage distinct from the inner cell mass of the mammalian blastocyst (Kelly et al., 1978). Early in embryo development, trophoblast functions to support formation of the blastocoel (Ducibella et al., 1975), and helps maintain a microenvironment suitable for the developing inner cell mass. In progression, trophoblasts perform dedicated functions to support survival of the embryo and fetus by establishing the critical extraembryonic components of the placenta (Mossman, 1937).

Placental development and morphology clearly differ among species particularly due to evolutionary pressures that remain poorly understood (Wildman et al., 2006; Garratt et al., 2013). The placental interface in cattle is the least invasive epitheliochorial type, with villous digitations between fetal and maternal tissues restricted to regions of the cotyledons (Björkman, 1969; Leiser and Kaufmann, 1994).

At present, the most basic knowledge and progress of trophoblast biology are from studies on mice (Simmons and Cross, 2005) and humans (Roberts and Fisher, 2011), both of which do not entirely represent the distinct morphological and functional features of bovine trophoblasts. Unlike mice and humans, the hatched bovine blastocyst remains free in the uterine fluid, and trophoblasts enter a phase of rapid proliferation and dramatic elongation that allow it to occupy sufficient surface for optimal attachment to maternal caruncles. This elongation is observed concomitant with gastrulation starting at gestational day 14, and the ‘filamentous’ embryo reaches the entire length of both uterine horns by gestational day 18–19 (Chang, 1952). During this period, trophoblasts of the bovine blastocyst are known to produce interferon-τ, a factor that ensures receptivity of the maternal endometrium by preventing a return to ovarian cyclicity (Roberts et al., 1992b). Ultimately, trophoblast cells differentiate, an event that is morphologically apparent first at the cotyledons, forming binucleate cells, and attaching to the caruncle by fusion with the epithelium forming trinucleate cells (Wooding, 1992). Beyond this association, functional differentiation leading to mechanisms of exchange between villous trophoblast and maternal blood is a topic that remains to be examined.

With interest in understanding physiological changes to the preimplantation embryo, there have been several studies examining transcription in the trophectoderm (Ozawa et al., 2012; Hosseini et al., 2015; Pfeffer et al., 2017), *in vitro* trophoblast cultures (Ushizawa et al., 2005; Ramos-Ibeas et al., 2014; Horcajo et al., 2017; Saadeldin et al., 2017), developmental stages of embryo elongation (Clemente et al., 2011; Hue et al., 2015) and differences attributed to embryo production methods (Betsha et al., 2013; Min et al., 2015; Velásquez et al., 2017). Despite the progress in describing transcriptional effects, the core characteristics of the bovine trophectoderm, trophoblast stem cells, and knowledge of genes and pathways regulating growth, development and function remain rudimentary. In this manuscript, we present the optimization of methods to culture primary blastocyst-derived bovine trophoblast colonies, and simultaneously profile the transcriptome and whole-cell proteome. We delineate these data using an atypical abundance-based functional classification for bioinformatics and physiological analysis of perceived relevance. We examine both integral components and those secreted into the blastocoel and/or uterine microenvironments. With early-pregnancy loss being a major concern in dairy cattle (Diskin and Morris, 2008; Wiltbank et al., 2016), our results on undifferentiated bovine trophoblast biology and core characteristics of bovine trophoblast stem cells represent a broad foundation for functional studies on early pregnancy and initiation of implantation in cattle.

## RESULTS AND DISCUSSION

Trophectoderm development during preimplantation stages is represented by a series of functional transitions concurrent with patterning of the embryo [reviewed in (Pfeffer and Pearton, 2012)]. In ungulates, rapid trophoblast proliferation during the period of embryo elongation is a striking feature that indicates that growth and patterning are regulated differently compared to other well-studied species like mice and humans. As undifferentiated trophoblasts self-renew and are sustained for regulated spatial and temporal differentiation to different components of the placenta, the term trophoblast stem cells has been used to describe *in vitro* cultures maintained in such a state (Tanaka et al., 1998). Although bovine trophoblast cell lines, CT-1 and CT-5 (Talbot et al., 2000), and BT-1 (Shimada et al., 2001) have been established, detailed profiling for defining trophectodermal features have not been performed for this species. In this study we describe the core characteristics of early stage blastocyst-derived trophoblasts that are of functional significance and describe hallmarks for the bovine self-renewing undifferentiated state.

### MEFs support bovine trophoblast attachment and growth

**Fig. 1.**
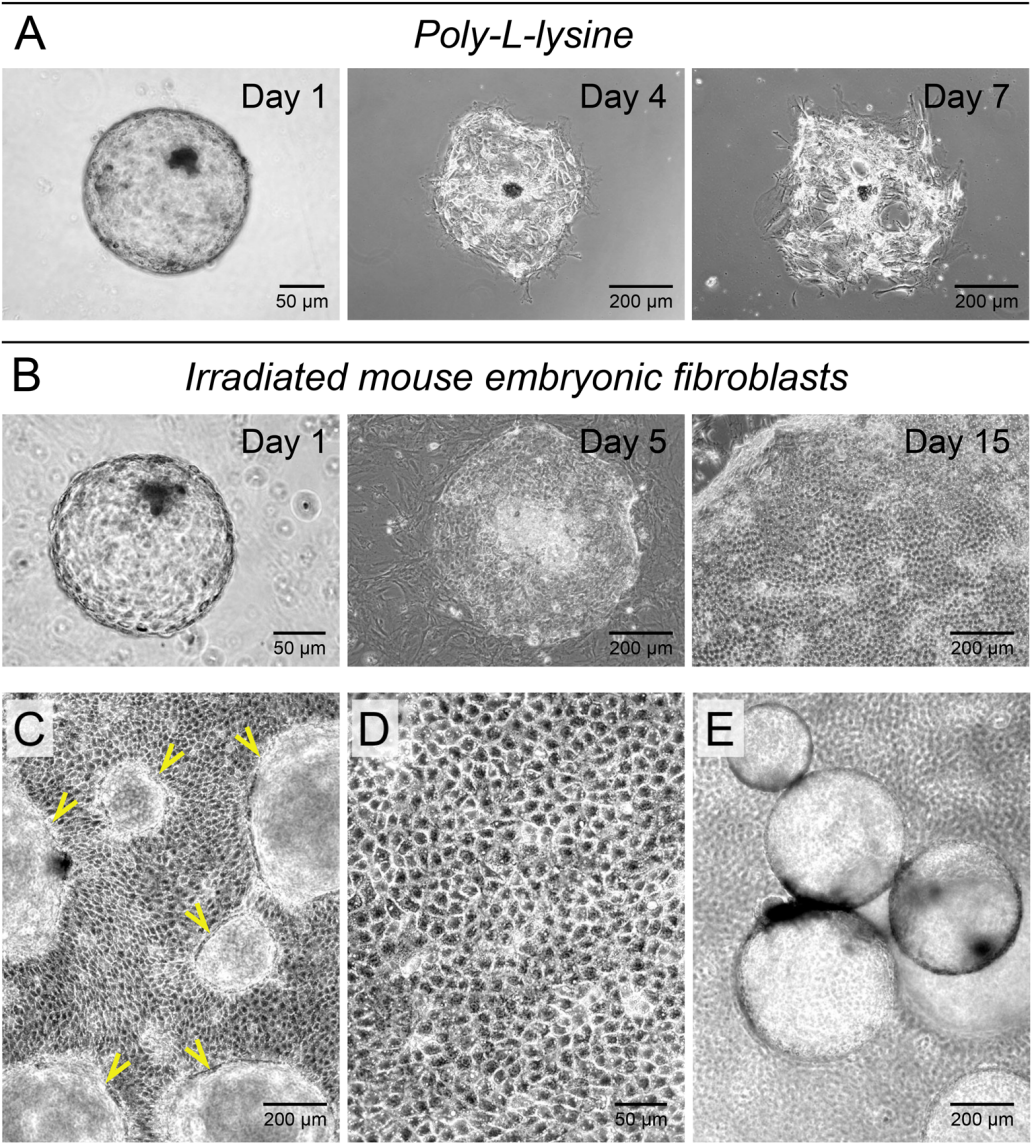
**Mouse embryonic fibroblasts support attachment and growth of bovine blastocyst-derived trophoblasts.** (A) Poly-L-lysine coated surfaces did not support bovine blastocyst attachment and trophoblast outgrowths. Of the blastocysts that attached, cells failed to expand and rapidly disintegrated. (B) Irradiated mouse embryonic fibroblast feeders (MEFs) allowed for blastocyst attachment and proliferation of the trophectoderm leading to colony formation. (C) Trophoblast colonies grew with limited basal attachments as sheets and formed numerous surface outpocketings (arrowheads) over time. (D) Proliferating trophoblast cells formed a characteristic polygonal cell sheet with prominent cell adhesions and resolvable cytoplasmic elements within. (E) As a result of pinch-offs from surface outpocketings, fluid-filled hollow trophoblast spheres analogous to the blastocyst-trophectoderm organization were frequently released from trophoblast colonies in culture.

*In vitro*-produced day-7 zona-free bovine blastocysts were used to determine conditions that would support attachment and establishment of blastocyst-derived trophoblast colonies. Attachment and colony formation failed when gelatin, poly-L-lysine or Matrigel^®^ were used as surface treatments; use of a layer of MEFs resulted in high levels of attachment and colony formation (Table 1, Fig. 1). Blastocysts attached in 1–2 days and formed outgrowths, which developed into ∼1 cm^2^ colonies by 15 days. Addition of FGF4 did not have an effect on blastocyst attachment and/or trophoblast colony formation; there was also no appreciable difference in colony size with and without FGF4 (not shown). Previous studies have demonstrated that FGF4 cannot be detected in MEF-conditioned medium (Sarkar et al., 2012).

**Table 1.**
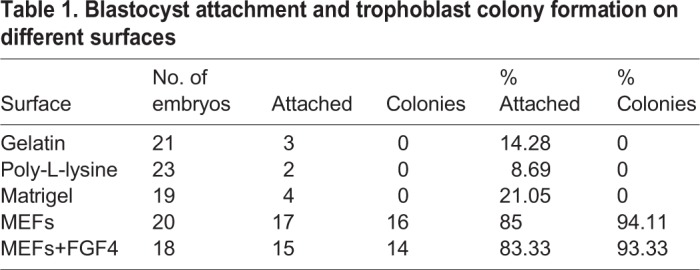
**Blastocyst attachment and trophoblast colony formation on different surfaces**

The resulting colonies on MEFs grew as sheets of proliferating cells with prominent cell adhesions and maintained minimal basal adhesions with tethering obvious toward the colony edges. Over time, trophoblast cultures showed numerous surface outpocketings and release of hollow trophoblast cysts homologous to the blastocyst-trophectoderm organization, called trophoblast vesicles or ‘trophocysts’ (Movie 1). Such 3-dimensional organization has been described for trophoblast stem cells from mice (Tarkowski and Wroblewska, 1967; Gardner et al., 1973; Rivron et al., 2018), primates (Summers et al., 1987) and humans (Weber et al., 2013; Nandi et al., 2018). In the bovine preimplantation embryo, this characteristic persists through elongation and has been previously demonstrated *in vitro* (Hashizume et al., 2006). The underlying reason could be that tight junctions between early trophectodermal cells present a diffusion barrier that allows for accumulation of fluid, a process similar to the formation of a blastocoel (Ducibella et al., 1975; Magnuson et al., 1978). Therefore, our early trophoblast cultures present characteristics of the trophectoderm. Trophocyst formation was also reported in feeder-free BT-1 cell line cultures (Shimada et al., 2001), indicating that certain characteristics can also be retained in long-term/immortalized trophoblast cultures.

### Morphology and functional characteristics of bovine trophoblasts in culture

**Fig. 2.**
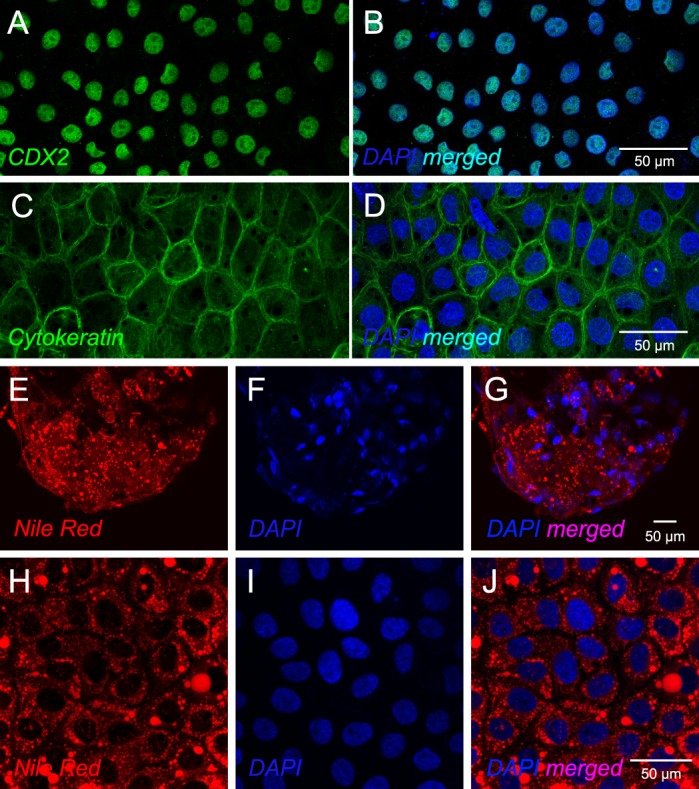
**Cultured bovine blastocyst-derived trophoblast colonies retain form and function of the trophectoderm.** (A,B) Trophoblast colonies were positive for CDX2, a transcription factor considered a marker for this lineage, known to play an important role in trophectoderm development. (C,D) Trophoblast colonies also showed existence of a complex network of cytokeratin, an arrangement that enables the trophectoderm to resist mechanical stress. (E–G) Cytoplasmic lipid droplets in the blastocyst-tropectodermal layer are abundant (stained by Nile Red), and indicative of cellular homeostasis linked to energy storage and lipid metabolism. (H–J) Lipid droplets were also prominent in cultured trophoblast colonies indicating maintenance of metabolic properties. For all panels, cell nuclei are counterstained with DAPI.

Sheets of blastocyst-derived trophoblast colonies on MEFs were formed of tightly packed cells with prominent cell adhesions and cytoskeletal elements. All cells in these trophoblast colonies were positive for CDX2, a core transcription factor responsible for trophectodermal development, and trophoblast stem cell self-renewal (Strumpf et al., 2005; Berg et al., 2011) (Fig. 2A,B). Overexpression of *Cdx2* in murine embryonic stem cells (ESCs) also forced their functional conversion to trophoblast stem cells (Niwa et al., 2005). Cytoskeletal organization in trophoblasts showed a consistent pattern indicated by the framework of cytokeratin (Fig. 2C,D). Prominent cytoplasmic lipid droplets were also observed in cultured trophoblasts indicating maintenance of metabolic properties similar to the bovine blastocyst trophectodermal layer that also shows cytoplasmic lipid droplets (Fig. 2E–J). These trophoblasts also expressed interferon-τ (IFNT; discussed below). Therefore, trophocysts that emerge from these colonies could be functionally comparable to trophoblast vesicles derived from elongating bovine blastocysts that could maintain the corpus luteum after uterine transfer to cyclic cows (Heyman et al., 1984).

### Trophoblast transcriptomics validated similarities to blastocyst-trophectoderm

**Fig. 3.**
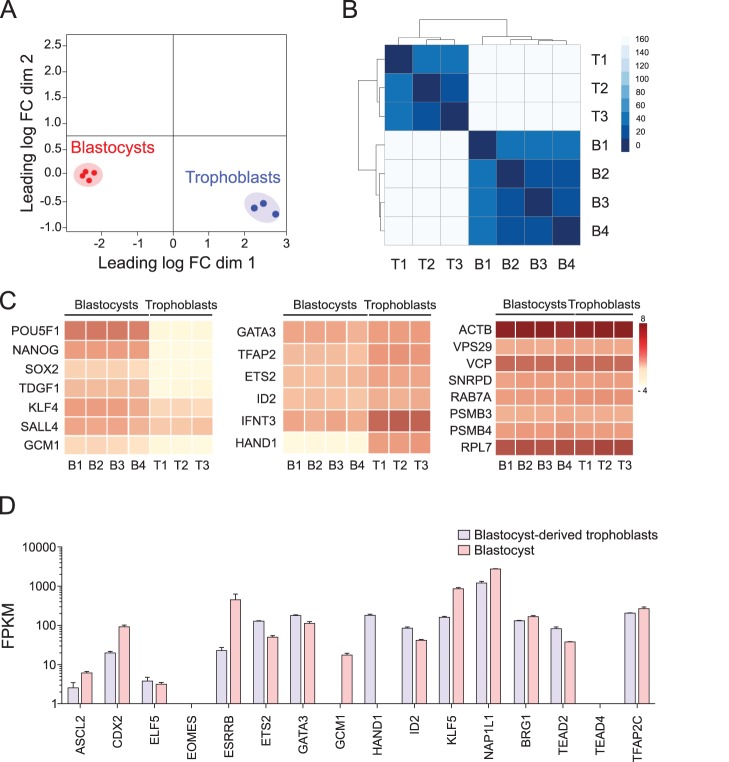
**Homogeneity within cultured bovine blastocyst-derived trophoblast colonies and trophoblast stem cells.** (A) Multidimensional scaling plot of blastocyst (*n*=4) and trophoblast (*n*=3) transcriptome datasets. Within each group, biological replicates clustered together indicating similarity in the gene expression profiles. (B) Heatmap of sample distance and unsupervised hierarchical clustering based on global gene expression showing distinct blastocyst and trophoblast colony datasets. Samples were clustered by Euclidean distance. (C) Heatmap representation highlighting similarities and distinctions in gene expression by trophoblast colonies compared to blastocysts. In trophoblast colonies, expression of pluripotency related genes was low, and trophoblast-specific genes were similar or higher than blastocysts. Gene expression for a variety of housekeeping genes were similar between trophoblasts and blastocysts. Scale log FPKM. (D) Expression of transcription factors that define trophoblast stem cells as reported for mice and humans, correlating day-7 blastocysts and blastocyst-derived trophoblasts (mean±s.e.m.).

RNA sequencing was performed to generate the transcriptome profile of *in vitro* cultured blastocyst-derived trophoblasts and was compared to the transcriptome of day-7 blastocysts. Consistency in gene expression profiles were confirmed across three independently generated trophoblast colonies, with a distinct clustering pattern when compared to day-7 blastocysts (Fig. 3A,B). On comparing transcript expression in trophoblast colonies with day-7 blastocysts, trophoblast-specific genes were found in both datasets; core pluripotency genes *POU5F1*, *NANOG* and *SOX2* associated with the inner cell mass were not expressed in trophoblast colonies (Fig. 3C). Comparison of transcription factors expressed in trophoblast stem cells as reported for mice and humans (Tanaka et al., 1998; Ohinata and Tsukiyama, 2014; Okae et al., 2018), showed consistencies and some deviations (Fig. 3D). Expression of *CDX2*, *ELF5*, *ID2*, *KLF5*, *ESRRB* and *TFAP2C*, considered critical transcription factors for trophoblast stem cells, was as expected. A primary deviation was that *EOMES*, also considered critical, was not expressed; we believe that this is a species-specific difference because expression of *EOMES* was also not observed in day-7 blastocysts (Fig. 3D). Lack of *EOMES* expression in bovine blastocysts was also indicated in previous studies (Hall et al., 2005; Ozawa et al., 2012). Two additional distinctions were: GCM1, considered a transcriptional indication of differentiation to syncytiotrophoblasts (Simmons et al., 2008; Matsuura et al., 2011; Lu et al., 2013; Zhu et al., 2017), was expressed in day-7 blastocysts but not in blastocyst-derived trophoblasts. *HAND1*, considered a transcription factor that promotes differentiation to trophoblast giant cells (Scott et al., 2000; Hughes et al., 2004), was expressed in blastocyst-derived trophoblasts but not in day-7 blastocysts (Fig. 3D). Expression of *HAND1* without *GCM1* in the blastocyst-derived trophoblasts, and expression of *GCM1* only in the day-7 blastocyst not only present a functional contradiction based on knowledge of stemness and differentiation in other species (Hughes et al., 2004; de Mestre et al., 2009), but also indicate that transcriptional regulation at the bovine blastocyst trophectoderm and the resulting trophoblast stem cells have species-specific distinctions.

### Trophoblast proteome showed overrepresentation of structural proteins

**Fig. 4.**
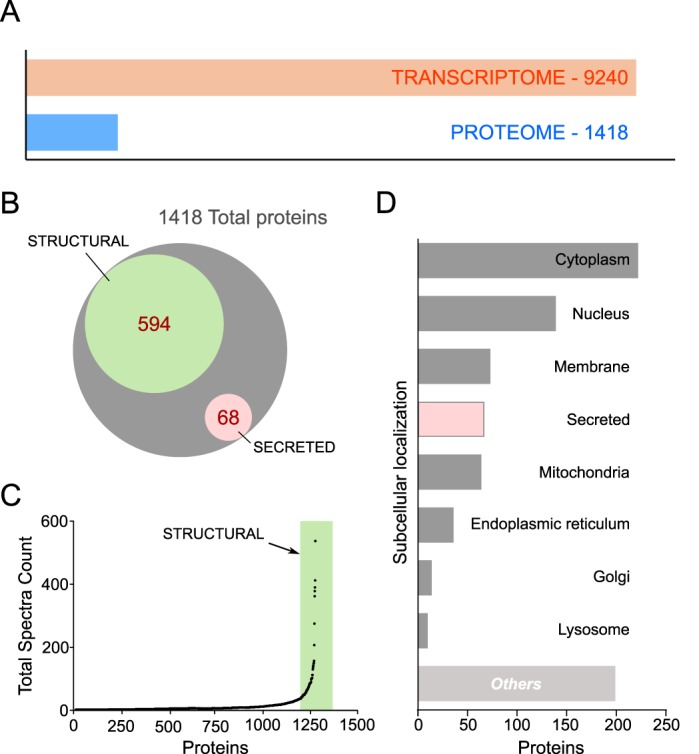
**Whole-cell proteomics indicated an overabundance of structural proteins in bovine blastocyst-derived trophoblasts.** (A) Comparison of total proteins identified with the total transcripts sequenced in blastocyst-derived trophoblasts. (B,C) Euler diagram and dot plot of spectra counts showing the disproportionate abundance of structural proteins in the proteomics dataset. (D) Subcellular classification of proteins identified in the trophoblast proteome after filtration using the algorithm to identifying secreted proteins.

Whole-cell proteomics detected only 1418 proteins (15.3% of the transcriptome) (Fig. 4A). Upon analysis we detected that skewed high abundance of structural elements (41.9% of proteins identified) might have masked the identification of low abundant proteins (Fig. 4B,C). This indicated that whole-cell proteomics was not fully representative of the entire functional features of these cells. Of the 1418 proteins, 68 were identified as secreted (Fig. 4D). We are not discussing these separately as most were also identified in the transcriptome. Notably, expression of trophoblast Kunitz-domain proteins (TKDPs) and pregnancy associated glycoproteins (PAGs) were prominent secreted elements identified in the proteomics. Full lists of proteins identified are provided as supplementary information (Table S1); raw data, mzML and scaffold results are available from the MassIVE proteomics repository (MSV000083135).

### Quantitative classification of trophoblast transcriptomics established prominent functional elements

**Fig. 5.**
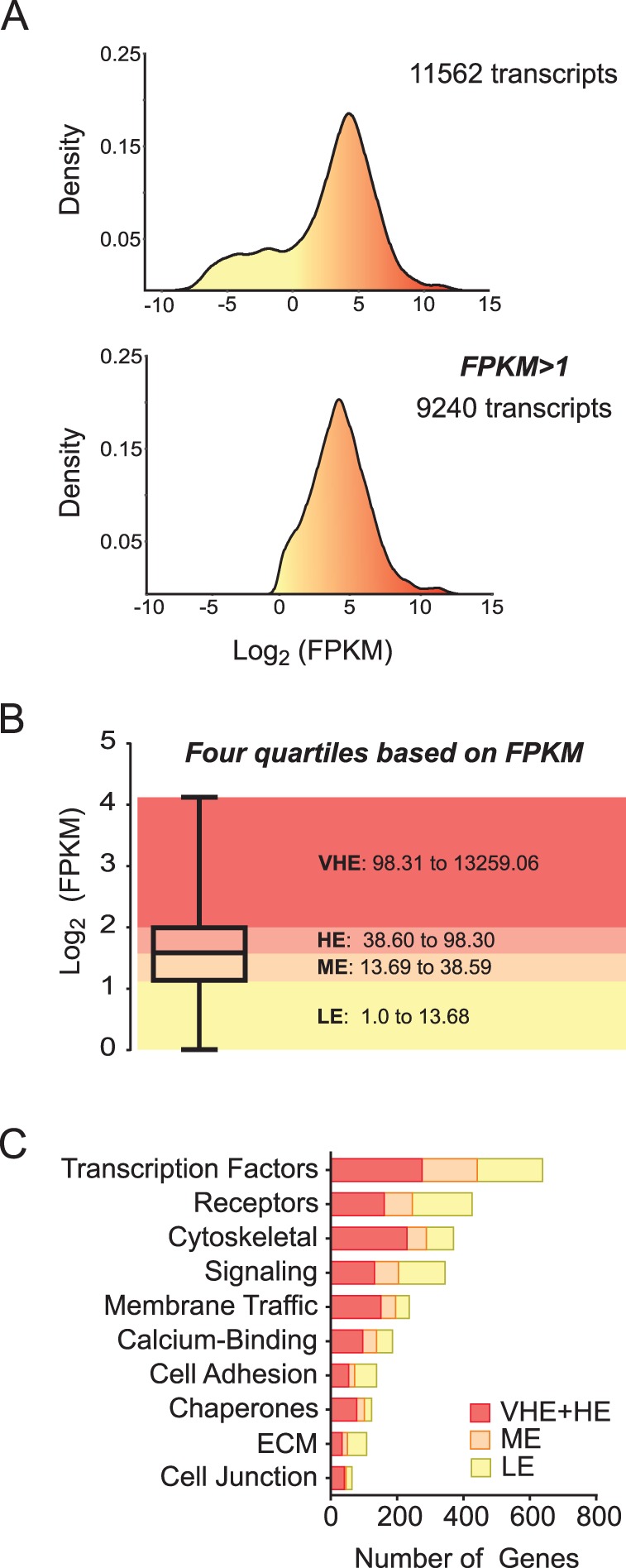
**Quantitative classification of gene expression based on gene ontology terms in bovine blastocyst-derived trophoblasts.** (A) Density histograms of gene expression levels from RNA Seq analysis of trophoblast cells before and after filtering for FPKM>1 threshold. (B) Classification of transcriptome into expression-based quartiles for functional analyses. Genes were classified based on FPKM into very highly expressed (VHE), highly expressed (HE), moderately expressed (ME) and low expressed (LE) groups. (C) GO terms assigned to transcripts (VHE, HE, ME and LE) showing relative distribution across molecular function terms.

Gene expression data from the trophoblast transcriptome were first filtered by selecting only transcripts that had FPKM>1 and eliminating ultra-low expression and false-positives (Fig. 5A). The resulting 9240 transcripts were then grouped into very high expression (VHE), high expression (HE), medium expression (ME) and low expression (LE) categories by delineating the distribution of absolute expression into four quartiles (Fig. 5B). This approach allowed for both combined and quantitatively separated analyses to provide varied thresholds in refining this dataset. Gene ontology (GO) terms were assigned to transcripts and lists generated for molecular function (Fig. 5C). The functional categories of relevance are highlighted below, and the full list including analyses and classifications is provided as supplementary information (Table S1); the complete RNA-seq datasets are also available through NCBI GSE (GSE122418).

#### Growth factors, cytokines and other secreted factors

Table 2 shows the complete list of GO: growth factors and cytokines together with other known factors of functional relevance in blastocyst-derived trophoblasts. The TKDPs constitute a placenta-specific family of proteins that exist only in ruminant ungulates and are expressed for short periods of time in the preimplantation embryo (MacLean et al., 2003). *TKDP4*, the only TKDP with high proteinase inhibitory activity (MacLean et al., 2004), was among the VHE group in blastocyst-derived trophoblasts. The PAGs represent a diverse family of proteins expressed exclusively by trophoblasts in patterns that vary with differentiation (Zoli et al., 1991; Xie et al., 1994; Roberts et al., 1995; Green et al., 2000). *PAG2*, *PAG11*, *PAG12* and *PAG8* were among the VHE group in blastocyst-derived trophoblasts. Similarly, interferon-τ 3 (*IFNT3*), a factor that ensures receptivity of the maternal endometrium by preventing a return to ovarian cyclicity (Roberts et al., 1992a,b) was also in the VHE group in blastocyst-derived trophoblasts. The secreted factors also contained a variety of specific receptor ligands. This list included factors such as *PDGF* (HE), *FGF2* (ME) and *IL6* (ME) that have been demonstrated to be important for maintaining pluripotency in other species (Nichols et al., 1994; Yoshida et al., 1994; Vallier et al., 2005; Wong et al., 2012), and others such as *FGF1* (VHE), *HDGF* (VHE), *VEGF* (VHE), *FGF2* (ME) and *BMP4* (ME) that are known to differentiate cells to specific lineages.

**Table 2.**
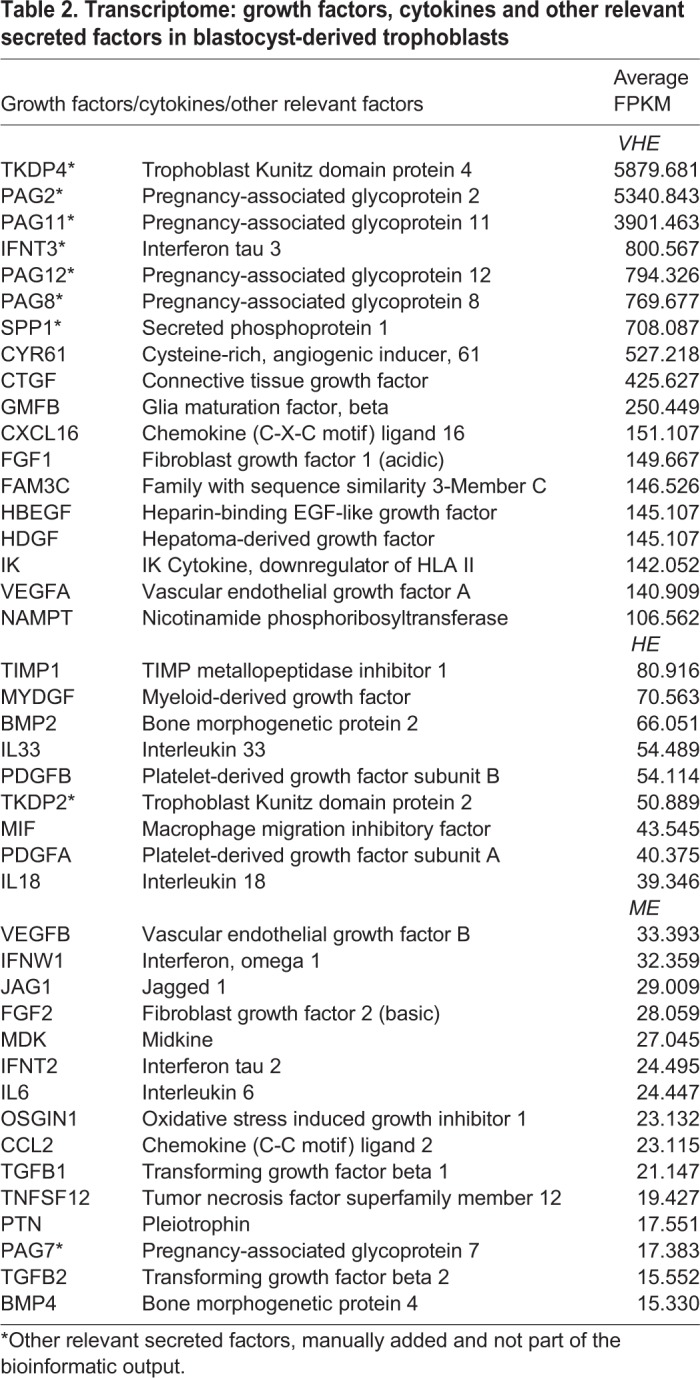
**Transcriptome: growth factors, cytokines and other relevant secreted factors in blastocyst-derived trophoblasts**

#### Structural elements

Table 3 shows selected list for GO: cytoskeletal elements in blastocyst-derived trophoblasts. Several of these transcripts were also identified by proteomics (Table S1). In addition to the actin and tubulin functional cytoskeletal network and associated proteins that maintain the dynamic state and vesicle/organelle transport, there was prominent presence of intermediate filaments, particularly cytokeratins which help these cells resist mechanical stress. Different cytokeratins have been reported in trophoblasts across different species (Jackson et al., 1980; Daya and Sabet, 1991). There was also presence of transcripts encoding a functional cohort of contractile elements such as myosin, tropomyosin and associated proteins. We speculate that contractions that occur during blastocyst hatching might be a myosin-driven feature. Presence of myosin has been previously reported in murine trophoblasts, and hypothesized to be associated with controlling invasion during implantation (Sobel et al., 1980). Trophoblasts also expressed Ezrin-Radixin-Moesin (ERM) transcripts/proteins that are known to organize signaling beneath the cell surface interfacing the extracellular environment and the cytoplasm (Neisch and Fehon, 2011). Specific transcripts encoding elements such as plakophilin (Chen et al., 2002) and testin (Coutts et al., 2003) that anchor focal adhesions to the cytoskeleton were also detected.

**Table 3.**
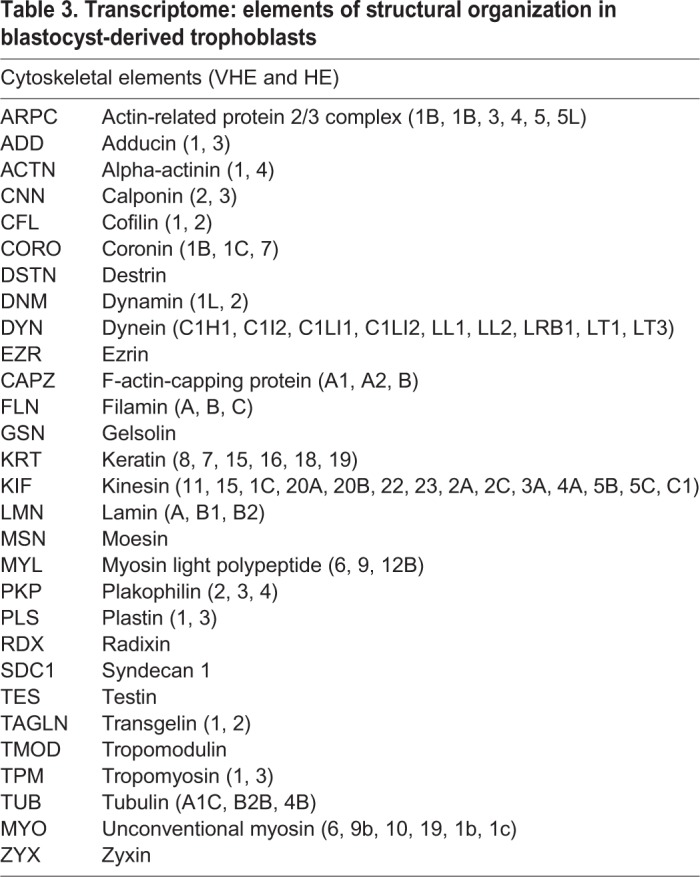
**Transcriptome: elements of structural organization in blastocyst-derived trophoblasts**

#### Extracellular organization

Tables 4 and 5 show selected lists for GO: extracellular matrix components and GO: cell junction and adhesion components respectively. Transcripts encoding three major extracellular components (fibronectin, laminin and collagen) that are also associated with a basement membrane were detected in blastocyst-derived trophoblasts. Previous studies have demonstrated that the above three play a role in adhesion and migration of cells (Martin et al., 1984; McCarthy et al., 1985). Transcripts encoding cell junctions were prominent with tight junctions (cadherins and claudins), gap junctions (gap junction beta 2, 6), signal triggers (integrins) and other stabilizing components. The existence of gap junction proteins in trophoblasts have been reported in rats (Grummer et al., 1996) and humans (Cronier et al., 2002); this suggests that cells of the trophectoderm could communicate as a syncytium. Beyond communication, it has been demonstrated in human trophoblasts that a protein kinase A-ezrin-gap junction alpha 1 signaling complex controls trophoblast fusion (Pidoux et al., 2014). In human trophoblasts, ezrin and E-cadherin expression were modulated by cytokines IL-1ß and TGF-ß1 (Karmakar and Das, 2004). A vast array of integrins that are expressed indicate signaling via ligand occupancy or by clustering alone (Akiyama, 1996; Vicente-Manzanares and Sánchez-Madrid, 2018). Integrin expression and its regulation have been studied in human trophoblasts (Burrows et al., 1993; Irving and Lala, 1995), and changes to the integrin profile has been observed during trophoblast invasion (Damsky et al., 1994). Integrins have also been detected in bovine trophoblasts of the placentome suggesting a role in functional attachment (Pfarrer et al., 2003).

**Table 4.**
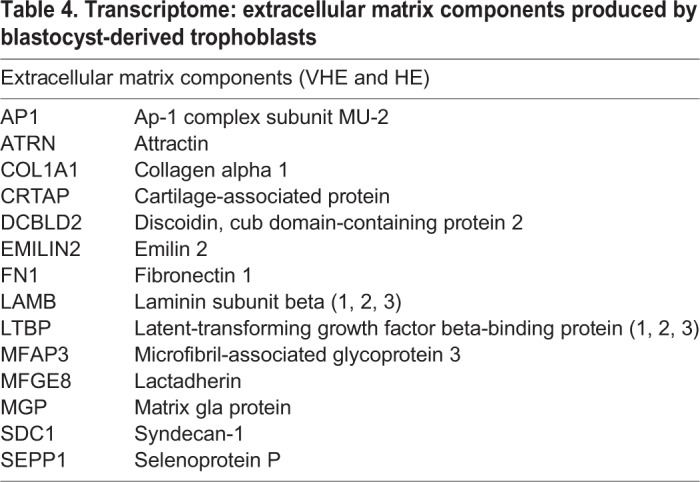
**Transcriptome: extracellular matrix components produced by blastocyst-derived trophoblasts**

**Table 5.**
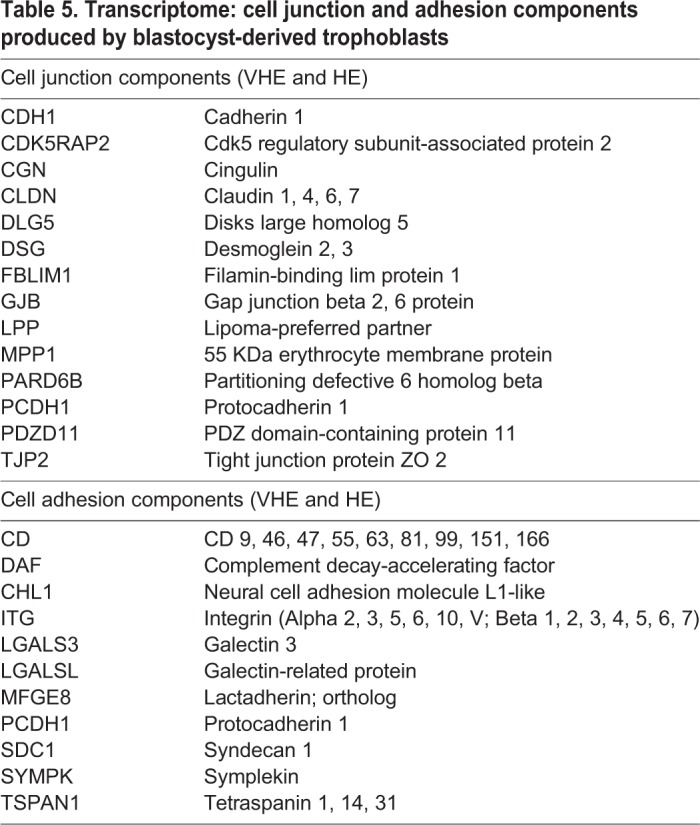
**Transcriptome: cell junction and adhesion components produced by blastocyst-derived trophoblasts**

#### Metabolic profile

For this analysis, we examined for specific transcripts across the different energy-generation systems using (Table 6). Importance of energy metabolism and changes to metabolic state that occur during differentiation has been highlighted in studies on human trophoblasts (Bax and Bloxam, 1997; Nadra et al., 2006; Pathmaperuma et al., 2010). Although our data do not quantitate the order of functional prominence in blastocyst-derived trophoblasts, they highlight the breadth of possibilities for metabolic functions. First, we find that *GLUT3* (*SLC2A3*) is the predominant glucose transporter in bovine trophoblasts with low to very low expression of *GLUT1* (*SLC2A1*) and *GLUT5* (*SLC2A5*). Initially identified as the neuronal glucose transporter, *GLUT3* is known to be highly expressed in neurons and has been classically considered as insulin insensitive (Nagamatsu et al., 1994; Olson and Pessin, 1996), meaning that they do not require insulin for translocation to the plasma membrane. *GLUT1* is consistently insulin sensitive (Ebeling et al., 1998). *GLUT3* has higher affinity for glucose than *GLUT1* and at least a fivefold greater glucose transport capacity (Simpson et al., 2008). This raises an interesting possibility that glucose uptake in trophoblasts may not be insulin-dependent. Transcripts encoding enzymes of glycolysis and gluconeogenesis were expressed in trophoblasts suggesting that these processes are active. Transcripts encoding enzymes in galactose metabolism feeding into glycolysis and pentose phosphate pathway were also expressed. Second, transcripts encoding components of lipid synthesis, transport, storage and metabolism were expressed at high levels in blastocyst-derived trophoblasts. Fatty acid synthase (*FASN*), scavenger receptor *CD36*, and low-density lipoprotein receptor (*LDLR*) were in the VHE group. Substantial fatty acid synthesis and release has been previously reported in human trophoblasts (Coleman and Haynes, 1987). The phenotype of the trophectoderm and blastocyst-derived trophoblast cells show abundant lipid droplets (Fig. 2) indicating that lipid accumulation could be a primary reserve for energy metabolism in these cells. There was also indication for active cholesterol synthesis with *HMGCR* in the VHE group, and the ability to generate pregnenolone (*CYP11A1*), but subsequent conversion to progesterone and estradiol. Third, all elements for TCA cycle and mitochondrial oxidative phosphorylation were also expressed in trophoblasts. *In vivo*, glucose and oxygen availability in the uterine fluid could be determinants of preimplantation metabolic status of the trophectoderm. Based on studies performed using human trophoblasts, metabolic adaptations can be quite distinct during trophoblast differentiation (Bax and Bloxam, 1997).

**Table 6.**
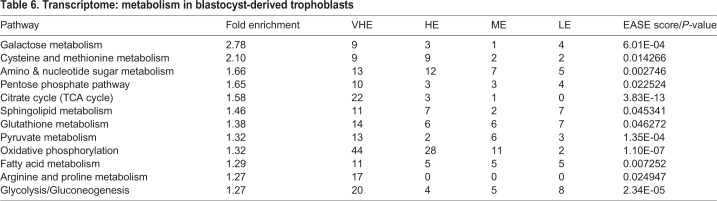
**Transcriptome: metabolism in blastocyst-derived trophoblasts**

#### Transcriptional profile

For this analysis, we generated a full list of transcription regulators present in bovine blastocyst-derived trophoblasts and then modeled the pathways that they represent (Table 7). Basic leucine zipper domain (*bZIP*) forms a large cohort of transcription factors with numerous downstream functions. It was observed as a prominent pathway predicted from expressed transcripts and it encompasses numerous factors with diverse functions. For example, *bZIP* transcription factors *ATF1* and cAMP response element binding (*CREB*) factor were found critical for blastocyst formation and survival in mice (Bleckmann et al., 2002); the same factors induce human chorionic gonadotrophin expression in human trophoblasts (Matsumoto et al., 1998; Knofler et al., 1999). Activin ß signaling, primarily through SMADs was observed. In murine trophoblast stem cells, activin signaling has been reported to maintain self-renewal (Zhu et al., 2015). SMADs are also the main signal transducers for the TGFß signaling pathway (Abdollah et al., 1997). The Toll gene in *Drosophila* is one of the key genes determining the developmental body plan (Anderson et al., 1985). It was subsequently rediscovered for its role in immunity, and mammalian Toll-like receptors are well studied in the context of pathogen defense (Akira and Takeda, 2004). In murine trophoblast stem cells, TLRs 1-6 were found to be expressed (Aikawa et al., 2014). We found *TLR2*, *TLR3* and *TLR6* expressed in bovine blastocyst-derived trophoblasts. Although innate immune functions relevant to invasive placentas have been suggested (Rose et al., 2011), presence in the bovine trophectoderm remains to be functionally examined. Active synthesis of ribosomal RNA (rRNA) transcripts was indicated by the enrichment of RNA polymerase I, perhaps an indication of proliferation. In other cell systems, increases in rRNA transcription increased proliferation and vice versa (Hayashi et al., 2014; Zhang et al., 2014).

**Table 7.**
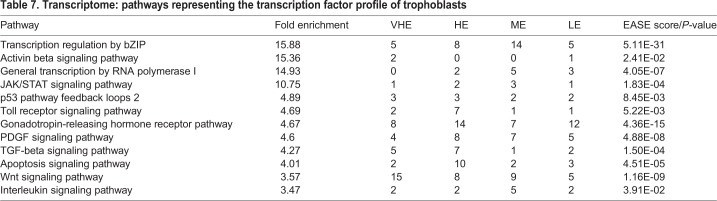
**Transcriptome: pathways representing the transcription factor profile of trophoblasts**

Signaling via the JAK/STAT signaling pathway (Darnell, 1997) was predicted to be active with expression of STAT1, STAT2 and STAT3 transcripts. In human trophoblasts, STAT signaling has been demonstrated to be involved in invasive differentiation (Fitzgerald et al., 2005; Poehlmann et al., 2005). In murine trophoblasts, leukemia inhibitory factor (LIF) mediated STAT3 signaling was found important for placental development and implantation (Cheng et al., 2001; White et al., 2007; Winship et al., 2015). Upstream, the major effects resulting from deletion of interleukin 6 signal transducer (IL6ST or gp130), a shared co-receptor for IL6 cytokines including LIF, are abnormal placental development and lethality (Yoshida et al., 1996). Deletion of STAT3 results in much earlier lethality (Takeda et al., 1997), as it is a broad downstream effector beyond LIF/IL6 signaling. PDGF signaling pathway as observed in bovine blastocyst-derived trophoblasts is also known to signal downstream via STAT (Sachsenmaier et al., 1999). Previous work on bovine embryos suggest beneficial effect for PDGF to bovine blastocyst development (Thibodeaux et al., 1993a,b). STATs are also downstream of interleukin signaling, as noted above for IL6-type cytokines (Heinrich et al., 2003). The p53 pathway that responds to genomic stress due to fidelity of DNA replication was also enriched in bovine blastocyst-derived trophoblasts. The p53-induced positive feedback loop promotes cell survival (Harris and Levine, 2005); this circuit communicates with other signaling pathways including WNT and apoptosis. Signaling in response to WNT has been implicated in expression of endogenous retrovirus-derived transcripts in bovine placentas (Sakurai et al., 2017). In human trophoblasts, WNT5a has been demonstrated to be critical for promoting proliferation and survival (Meinhardt et al., 2016). Signals for apoptosis converge from multiple survival and lack/deficiency of developmental factors. Enrichment of gonadotropin-releasing hormone (GnRH) receptor pathway was evident in the blastocyst-derived trophoblasts. GnRH has been observed in human placentas throughout gestation (Khodr and Siler-Khodr, 1980; Chou et al., 2004), and distinct from pituitary gonadotrophs, GnRH receptor is also present in human placentas (Lin et al., 1995; Cheng et al., 2000). Functionally, GnRH receptor signaling has been reported to induce trophoblast invasion (Liu et al., 2009). There are no previous reports on GnRH receptor and associated functions for bovine trophoblasts.

### Conclusion

Understanding the bovine trophectoderm helps interpret important developmental functions leading to pregnancy success in cattle. This study represents a comprehensive examination of functional and defining characteristics of bovine blastocyst-derived trophoblasts interpreted with the current state of understanding. Our results and databases provide a timeless foundation/reference for future functional studies for both advancing basic science and towards making improvements to cattle reproduction technologies.

## MATERIALS AND METHODS

### *In vitro* embryo production

Protocol for *in vitro* production of bovine embryos was as previously described (Negrón-Pérez et al., 2017). In brief, follicles measuring 2–10 mm were sliced to obtain cumulus oocyte complexes (COCs) from ovaries collected at the abattoir (Central Beef Packing Co., Center Hill, USA). COCs with at least one complete layer of compact cumulus cells were selected, washed in oocyte collection medium and placed as groups of 10 in 50 μl drops of oocyte maturation medium overlaid with mineral oil. The COCs were allowed to mature for 20–22 h in a humidified atmosphere of 5% CO_2_ at 38.5°C. After maturation, COCs were placed as groups of 50/well in four-well plates containing 425 μl of *In Vitro* Fertilization - Tyrode's Albumin Lactate Pyruvate (IVF-TALP) medium (Caisson Labs), and 20 μl of 0.25 mM hypotaurine, 25 μM epinephrine and 0.5 mM penicillamine in 0.9% NaCl (w/v). Semen from frozen-thawed straws from three bulls were pooled, purified with ISolate^®^ [Irvine Scientific; 50% (v/v) and 90% (v/v)], and diluted to a final concentration in the fertilization dishes of 1×10^6^/ml. Fertilization was allowed to proceed for 8–9 h in a humidified atmosphere of 5% CO_2_ at 38.5°C. After fertilization, putative zygotes were denuded of cumulus cells by vortexing in 100 μl hyaluronidase (1000 U/ml in approximately 0.5 ml HEPES-TALP), and cultured in groups of 25–30 in 50 μl synthetic oviduct fluid-bovine embryo 2 (SOF-BE2) in a humidified atmosphere of 5%, 5% and 90% (v/v) of CO_2_, O_2_ and N_2_, respectively, at 38.5°C. Embryos that developed to blastocysts at day 7 after insemination were used for trophoblast cultures.

### Attachment and growth conditions for primary trophoblast culture

Primary culture conditions for *in vitro* attachment and trophoblast growth were tested using zona removed or hatched day 7–8 blastocysts. Zona removal was performed using Pronase^®^ protease (0.1%, Sigma-Aldrich). As a base medium for testing different substrates, we used Dulbecco's modified eagle medium and M199 medium (1:1 ratio), containing 15% fetal bovine serum with added non-essential amino acids supplement and penicillin-streptomycin. All incubation was performed at 37°C in an atmosphere of 5% CO_2_. We evaluated the efficacy of coating with gelatin (2%, Sigma-Aldrich), poly-L-lysine (0.01%, Sigma-Aldrich), Matrigel^®^ (0.5 mg/cm^2^, Corning), or growing over an irradiated mouse embryonic fibroblast (MEF) feeder layer on blastocyst attachment and formation of trophoblast colonies. In conditions that supported trophoblast outgrowths, we also tested the effect of fibroblast growth factor 4 (FGF4; 25 ng/ml, GoldBio), that has been shown to promote growth of murine trophoblast stem cells (Tanaka et al., 1998). Outgrowth/colony for each embryo was allowed to expand to approximately 1 cm^2^ before collection for use in experiments. Images were acquired using either a DFC365FX camera in M80 stereo or an ICC50HD camera in DMIL inverted microscopes (Leica).

### Immunocytochemistry

To enable imaging, trophoblast outgrowths were grown on glass coverslips. Trophoblasts were fixed with 4% formaldehyde for 15 min at room temperature. Fixed cells were then permeabilized with 0.1% Triton X-100 in phosphate buffered saline (PBS) for 1 min and blocked using 5% normal goat serum for 30 min. Coverslips were subsequently incubated with a mouse monoclonal anti-cytokeratin antibody (Cell Signaling Technology; clone C11) or with an affinity-purified mouse monoclonal antibody against Caudal type homeobox 2 (CDX2; BioGenex, Cat # AM392) for 1 h. Coverslips were then washed three times using PBS and incubated with Alexa Fluor 488 conjugated anti-mouse Fab’ fragments for 30 min, washed again with PBS, counterstained/mounted with 4′,6-diamidino-2-phenylindole (DAPI) containing Prolong Gold reagent (Life Technologies). For staining lipid droplets, fixed trophoblasts were stained with 10 µg/ml Nile Red (Life Technologies) for 45 min followed by washing coverslips and mounting as described above. Images were acquired using a Meta 510 confocal microscope (Zeiss).

### Trophoblast transcriptomics

Trophoblast colonies were collected and total RNA was extracted using RNAqueous micro kit (Thermo Fisher Scientific) as three independent collections. Integrity was checked using the Bioanalyzer 2100 (Agilent Technologies), mRNA was isolated using poly(A) capture, fragmented and cDNA library construction was performed using TruSeq stranded total RNA sample preparation kit (Illumina). Samples with unique bar code sequences were pooled for sequencing by synthesis to obtain short single reads on a HiSeq4000 (Illumina). Reads were aligned to the bovine genome (UMD3.1) (Elsik et al., 2016) using Tophat (version 2.0.9) (Kim et al., 2013). Raw count for each gene was estimated with BioConductor (EdgeR version 3.18.1), package using BAM files. Multidimensional scaling (MDS) plot was generated using the plotMDS function of edgeR after normalization using the trimmed mean of M-values (TMM) method (Robinson and Oshlack, 2010). For unsupervised hierarchical clustering analysis, the R function ‘dist’ was used to calculate the Euclidean distance between the samples on rlog-transformed data (a pseudo count value of 1 FPKM was added to nulls). Heatmap.2 (gplots package in edgeR) was used to visualize the comparison.

### Trophoblast proteomics

For whole-cell proteomics, trophoblast colonies were lysed and directly solubilized using 6 M urea in 50 mM ammonium bicarbonate. Dithiothreitol (DTT) was added to a final concentration of 5 mM and samples were incubated for 30 min at 37°C. Subsequently, 20 mM iodoacetamide (IAA) was added to a final concentration of 15 mM and incubated for 30 min at room temperature, followed by the addition of 20 μl DTT to quench the IAA reaction. Lys-C/trypsin (Promega) was used at a 1:25 ratio (enzyme:protein) and incubated at 37°C for 4 h. Samples were then diluted to <1 M urea by the addition of 50 mM ammonium bicarbonate and digested overnight at 37°C. The following day, samples were desalted using C18 Macro Spin columns (Nest Group) and dried down by vacuum centrifugation. LC separation was done on a Proxeon Easy-nLC II HPLC (Thermo Fisher Scientific) with a Proxeon nanospray source. The digested peptides were reconstituted in 2% acetonitrile/0.1% trifluoroacetic acid and 10 μl of each sample was loaded onto a 100 μm×25 mm Magic C18 100 Å 5 U reverse phase trap where they were desalted online before being separated on a 75 μm×150 mm Magic C18 200 Å 3 U reverse phase column. Peptides were eluted using a gradient of 0.1% formic acid and 100% acetonitrile with a flow rate of 300 nl/min. A 120-min gradient was run with 5% to 35% acetonitrile over 100 min, 35% to 80% acetonitrile over 10 min, 80% acetonitrile for 2 min, 80% to 5% acetonitrile over 5 min, and finally held at 5% acetonitrile for 5 min. Mass spectra were collected on an Orbitrap Q Exactive mass spectrometer (Thermo Fisher Scientific) in a data-dependent mode with one MS precursor scan followed by 15 MS/MS scans. A dynamic exclusion of 5 s was used. MS spectra were acquired with a resolution of 70,000 and a target of 1×10^6^ ions or a maximum injection time of 20 ms. MS/MS spectra were acquired with a resolution of 17,500 and a target of 5×10^4^ ions or a maximum injection time of 250 ms. Peptide fragmentation was performed using higher-energy collision dissociation (HCD) with a normalized collision energy (NCE) value of 27. Unassigned charge states as well as +1 and ions >+5 were excluded from MS/MS fragmentation. Scaffold (version 4.2.0, Proteome Software Inc., Portland, USA) was used to validate MS/MS based peptide and protein identifications.

### Bioinformatics

For identifying secreted proteins, the proteome dataset was subjected to analysis for predicting candidates that are secreted via the classical cell secretory pathway as previously described (Pillai et al., 2017). First, we used SignalP v4.1 (http://www.cbs.dtu.dk/services/SignalP/) (Petersen et al., 2011) to examine N-terminal sequence motifs directing proteins to the secretory pathway; in tandem, we used TargetP v1.01 (www.cbs.dtu.dk/services/TargetP) (Emanuelsson et al., 2007) to refine this dataset by removing proteins destined for the mitochondria. The resulting list of candidates was further refined using Phobius (http://phobius.sbc.su.se/) (Käll et al., 2004), to eliminate integral membrane proteins that contained transmembrane regions. In this overall analysis, candidate proteins were considered secreted if they contained an N-terminal secretory sequence, did not traffic to the mitochondria, and lacked transmembrane regions.

For functional categorization of transcripts, the transcriptome dataset was organized by assigning gene ontology (GO) terms through PANTHER (protein analysis through evolutionary relationships) classification system (Thomas et al., 2003; Mi et al., 2017). Gene lists for the different expression groups (VHE, HE, ME and LE) were given as input. The PANTHER statistical overexpression test was used to identify GO annotations or pathways were overrepresented in comparison to a reference list, and a *P*-values are calculated based on expected values in the reference (Mi et al., 2013). We also performed gene enrichment and functional annotation analysis using DAVID 6.8 (database for annotation, visualization and integrated discovery) that integrates evaluation and prediction of metabolic pathways of the KEGG (Kyoto encyclopedia of genes and genomes) database (Kanehisa et al., 2016). A combined list of transcripts under VHE, HE, ME and LE were submitted. Fisher exact statistics measured input genes highly associated with functional groups providing a Fisher Exact Probability Value (called EASE score) for fold enrichment and assessment of significance. Results were visualized in KEGG Pathways. For additional functional evaluation of transcript data, identified genes and proteins were also analyzed using Ingenuity^®^ pathway analysis (IPA, Qiagen) to model and interpret biological significance of identified components (Krämer et al., 2014).

## Supplementary Material

Supplementary information
