# Supplementary material for: Physiological profile of undifferentiated bovine blastocyst-derived trophoblasts
Source: Biol Open. 2019 Apr 5;8(5):bio037937. doi: 10.1242/bio.037937 (PMC6550082; doi:10.1242/bio.037937)
Supplement: Supplementary information [file biolopen-8-037937-s1.pdf]

**Table S1.** Transcriptome and proteome information for undifferentiated bovine blastocyst derived trophoblasts.

(**Sheet 1**) Transcriptome: Complete list of transcripts identified.

(**Sheet 2, 3, 4, 5**) Transcriptome: Full list of genes expressed, classified based on transcript abundance as very high expression (VHE), high expression (HE), medium expression (ME) and low expression (LE) respectively.

(**Sheet 6**) Transcriptome: Transcription factors.

(**Sheet 7**) Transcriptome: Receptors.

(**Sheet 8**) Transcriptome: Ion channels.

(**Sheet 9**) Transcriptome: Enzymes.

(**Sheet 10**) Transcriptome: Peptidases.

(**Sheet 11**) Transcriptome: Cytoskeletal elements.

(**Sheet 12**) Transcriptome: Cell adhesion molecules.

(**Sheet 13**) Transcriptome: Cell junction proteins.

(**Sheet 14**) Proteome: Complete list of proteins identified.

(**Sheet 15**) Proteome: Secreted proteins classified based on bioinformatics.

Click here to download Table S1

Biology Open • Supplementary information

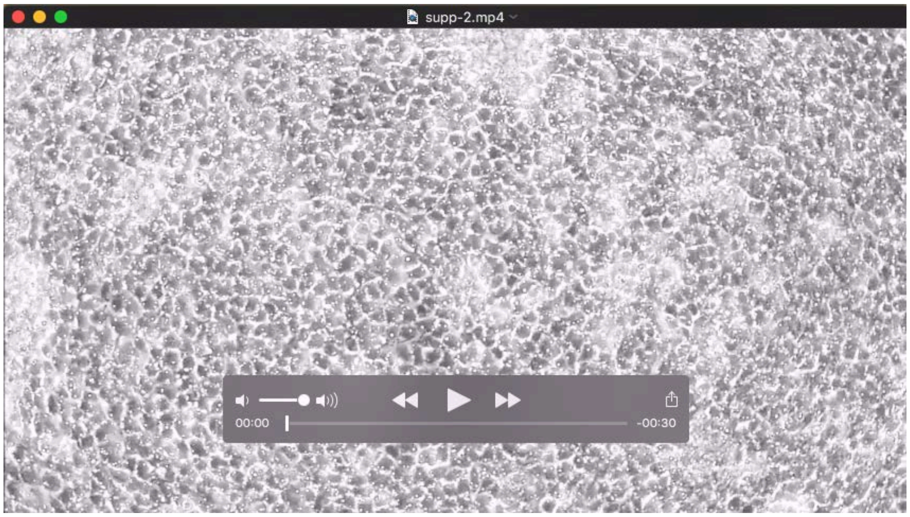


**Movie 1.** Morphological appearance of cultured bovine undifferentiated trophoblasts. Tightly packed sheet of proliferating cells with prominent cell adhesions that show spontaneous formations surface outpocketings, that are often released as hollow trophoblast cysts or ‘trophocysts’.
